# Immune Metabolism of IL-4-Activated B Cells and Th2 Cells in the Context of Allergic Diseases

**DOI:** 10.3389/fimmu.2021.790658

**Published:** 2021-12-01

**Authors:** Yen-Ju Lin, Alexandra Goretzki, Stefan Schülke

**Affiliations:** Molecular Allergology, Paul-Ehrlich-Institut, Langen, Germany

**Keywords:** allergies, metabolism, glycolysis, immune metabolism, oxidative phosphorylation, fatty acid oxidation (FAO), Warburg metabolism

## Abstract

Over the last decades, the frequency of allergic disorders has steadily increased. Immunologically, allergies are caused by abnormal immune responses directed against otherwise harmless antigens derived from our environment. Two of the main cell types driving allergic sensitization and inflammation are IgE-producing plasma cells and Th2 cells. The acute activation of T and B cells, their differentiation into effector cells, as well as the formation of immunological memory are paralleled by distinct changes in cellular metabolism. Understanding the functional consequences of these metabolic changes is the focus of a new research field termed “immune metabolism”. Currently, the contribution of metabolic changes in T and B cells to either the development or maintenance of allergies is not completely understood. Therefore, this mini review will introduce the fundamentals of energy metabolism, its connection to immune metabolism, and subsequently focus on the metabolic phenotypes of IL-4-activated B cells and Th2 cells.

## Introduction

Belonging to hypersensitivity disorders, allergies are abnormal, Th2-biased immune responses directed against otherwise harmless antigens derived from our environment. In susceptible individuals, contact with certain allergens results in the activation, differentiation, and proliferation of allergen-specific Th2 cells. Activated allergen-specific Th2 cells in turn produce IL-4, IL-5, IL-9, and IL-13 which can promote expansion, maturation, and/or functional activation of inflammatory cells, including mast cells, eosinophils, and allergen-specific B cells. These activated B cells in turn undergo class switch recombination of immunoglobulin genes to produce allergen-specific IgE antibodies (1). Finally, allergen-specific IgE antibodies mediate allergic inflammation by FcϵRI-mediated activation of mast cells, basophils, and perhaps eosinophils.

Over the last decades, the frequency of allergic disorders has steadily increased. Currently, IgE-mediated type I allergies affect more than 25% of the population in developed countries (2, 3), making them an important health and economic problem.

As described above, type I allergies arise from complex interactions between different types of immune cells. Hereby, activated immune cells undergo extensive changes in phenotype and function to fulfill their effector functions. Activation, differentiation, proliferation, migration, and mounting of effector responses all require metabolic reprogramming. This connection between immune cell function and cellular metabolic state is investigated in the relatively new research field termed “immune metabolism”.

Recent progress in basic research has repeatedly pointed out that immune cell activation and effector function are tied to distinct changes in the metabolism of the respective cell (4, 5). Here, changes in both overall metabolic activity and phenotype not only fulfill the rapidly increasing energy demand of activated cells but are also used to synthesize crucial immune effector molecules (e.g. prostaglandins, nitric oxide (NO), reactive oxygen species (ROS), or itaconate [reviewed in (6)].

Immune metabolism is also of relevance in allergic diseases (7). For example, Obeso et al. reported the progression to severe profilin allergic phenotypes to be characterized by alterations in platelet function, reduced protein synthesis, and the switch to glycolytic Warburg metabolism (8).

We recently summarized the contribution of immune metabolism to the activation and effector function of the innate immune cells involved in allergic reactions (7). Besides innate immune cells, T and B cells are also indispensable for the induction, maintenance, and resolution of allergic responses.

While changes in T and B cell metabolism clearly contribute to their activation, differentiation, and effector function [reviewed in (9–11)], currently, the contribution of metabolic changes in T and B cells to either the development or maintenance of allergies is not completely understood. Therefore, this mini review will briefly introduce the field of immune metabolism and subsequently focus on the metabolism of IL-4-activated B cells and Th2 cells.

## Immune Metabolism Is Critically Important for Immune Cell Effector Function

Glucose, fatty acids, and amino acids are the main cellular energy sources. Exposure of immune cells to different stimuli may require these cells to prioritize the biosynthesis of certain molecules or ROS over the simple production of energy in the form of ATP (12). To allow for this flexibility, cellular metabolism is a complex network of interconnected catabolic and anabolic pathways, most of which converge at the mitochondrion (13).

**Figure 1:**
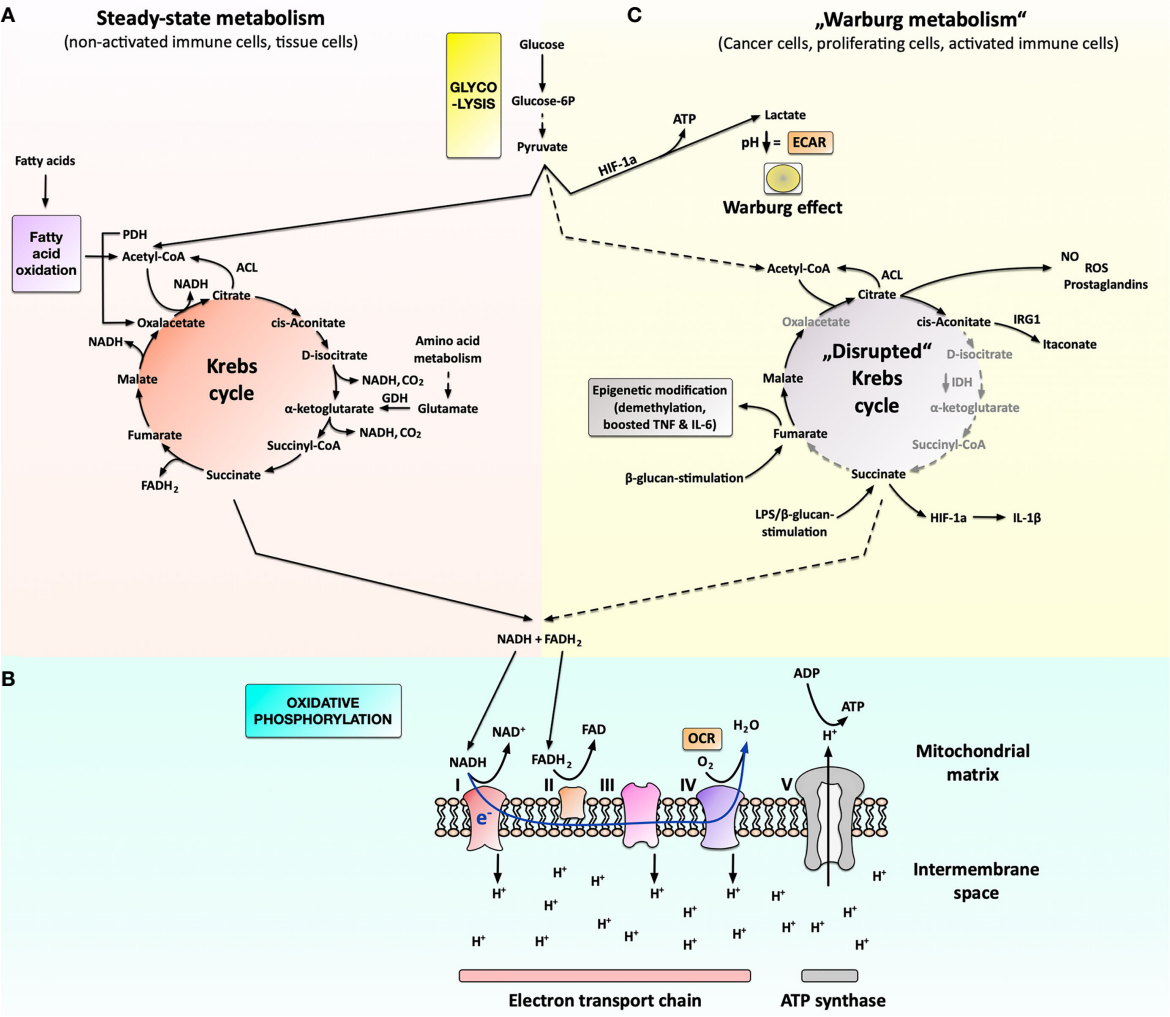
Cellular metabolism under steady-state conditions and metabolic changes associated with cancer cells, proliferating cells, or activated immune cells. Under steady-state conditions **(A)**, cells take up glucose from their medium and metabolize it to pyruvate in a cytoplasmic process called glycolysis. Pyruvate is subsequently imported into the mitochondria, where it is used in the Krebs cycle to generate the reduction equivalents NADH and FADH_2_. Besides glucose, the Krebs cycle can also be replenished by acetyl-CoA derived from fatty acid oxidation (FAO) or glutamate derived from amino acid metabolism. NADH and FADH_2_ generated in the Krebs cycle are then used in the oxidative phosphorylation to generate energy **(B)**. Here oxygen is used as the terminal electron acceptor in the electron transport chain (consisting of complexes I through IV) to generate a proton gradient over the inner mitochondrial membrane (measured as oxygen consumption rate (OCR)). This proton gradient drives the generation of ATP *via* the ATP-synthase complex. Under certain conditions, cancer cells, strongly proliferating cells, or activated immune cells switch their metabolism to predominantly produce lactate from glucose instead of fully oxidizing glucose into CO_2_ in the mitochondrion **(C)**. The produced lactate is secreted from the cell, leading to an extracellular pH decrease that can be measured as extracellular acidification rate (ECAR). Moreover, the lack of pyruvate results in a “disrupted” Krebs cycle, which the activated immune cells use to generate important biosynthetic intermediates needed for immune cell effector function such as prostaglandins, NO, ROS, or itaconate. Additionally, fumarate and acetyl-CoA (generated, for example, from citrate *via* the ATP-citrate lyase, ACL) can be used for epigenetic modification. For more information see main text. FAO, fatty acid oxidation; ROS, reactive oxygen species; NO, nitric oxide; FAS, fatty acid synthesis; PDH, pyruvate dehydrogenase; ACL, ATP-citrate lyase; GDH, glutamate dehydrogenase; IDH, isocitrate dehydrogenase; IRG1, immune-responsive gene 1; HIF-1a, hypoxia-inducible factor 1a; OCR, oxygen consumption rate; ECAR, extracellular acidification rate; I to IV, complex I to IV.

Under normal metabolic conditions, glucose (a C6 body) is converted *via* a multi-step pathway termed glycolysis into two molecules of pyruvate (C3 bodies) (**Figure 1A**). In the mitochondrion, pyruvate is metabolized into acetyl-CoA by the enzyme pyruvate dehydrogenase (PDH) (**Figure 1A**), which is used to drive the Krebs cycle (**Figure 1A**).

Besides pyruvate, the Krebs cycle can also be replenished by acetyl-CoA derived from fatty acid oxidation (FAO) as well as α-ketoglutarate generated from glutamate (derived from amino acid metabolism) (**Figure 1A**).

Ultimately, pyruvate, fatty acids, or amino acids degraded *via* the Krebs cycle result in the generation of both CO_2_ and energy in the form of the reduction equivalents NADH and FADH_2_ (**Figure 1A**) (10, 11). In a process termed oxidative phosphorylation (OxPhos), these reduction equivalents are subsequently transferred to the terminal electron acceptor oxygen (O_2_) to generate energy at the inner membrane of the mitochondrion *via* the ATP synthase complex (**Figure 1B**) (6). Therefore, OxPhos strongly depends on the availability of exogenous oxygen and can be quantified *via* the cellular oxygen consumption rate (OCR) (**Figure 1B**).

However, under certain conditions (see below), cells can switch to predominantly producing lactate from pyruvate (**Figure 1C**). Mechanistically, this shift towards lactate production is caused *via* the hypoxia-inducible factor 1a (HIF-1a)-dependent up-regulation of different pyruvate dehydrogenase kinase (PDHK) isoforms (**Figure 1C**) (14). The generated lactate cannot be further metabolized inside the cell and is subsequently secreted, acidifying the extracellular environment (**Figure 1C**) (15).

Lactate generation in eukaryotic cells is typically observed under anaerobic conditions but can also occur as “aerobic glycolysis” in cancer cells under conditions of adequate oxygen supply (16–18). First detected by Otto Warburg in cancer cells, “aerobic glycolysis” is therefore known as the “Warburg effect” (**Figure 1C**) (18). Experimentally, the Warburg effect can be detected by either measuring lactate levels in the cells supernatant, quantifying the yellow coloring in phenol red containing culture media optically, or determining the extracellular acidification rate (ECAR) (**Figure 1C**).

Recent research has shown that the Warburg effect not only occurs in cancer cells, but also in other cells characterized by a high energy demand, such as strongly proliferating cells or activated immune cells (15, 19).

The increased rates of aerobic glycolysis are used to both fulfill the rapid energy demands of activated immune cells and to provide certain Krebs cycle intermediates critically needed in activated and proliferating immune cells (19–23). For example, the predominant cytoplasmic metabolization of pyruvate into lactate results in a shortage of pyruvate which is usually fed into the mitochondrial Krebs cycle. This results in an arrest of the Krebs cycle at certain steps, which is termed a “disrupted” Krebs cycle (**Figure 1C**) (7, 24, 25).

In this context, a reduced expression of the enzyme isocitrate dehydrogenase (IDH), which normally converts D-isocitrate to α-ketoglutarate (26), was shown to result in the accumulation of the Krebs cycle intermediates citrate, cis-aconitate, and D-isocitrate facilitating the generation of important immune effector molecules such as prostaglandins, NO, ROS, or itaconate (**Figure 1C**) [reviewed in (6)]. Moreover, accumulation of fumarate upon lipopolysaccharide (LPS) re-stimulation of β-glucan-trained macrophages was reported to boost TNF- and IL-6-secretion *via* epigenetic modification (27). In addition, accumulation of succinate in LPS-stimulated macrophages was shown to promote glycolysis and IL-1β production *via* the stabilization of HIF-1a (**Figure 1C**) [reviewed in (24)].

Therefore, the metabolic shift towards Warburg metabolism allows activated immune cells to quickly produce the energy needed for their respective effector function and efficiently function in the oxygen-deprived microenvironment of the acutely inflamed/infected tissue. Moreover, the “disrupted” Krebs cycle is used to form important immune effector molecules.

### Immune Metabolic Changes Associated With the Activation of B and T Cells

**Figure 2:**
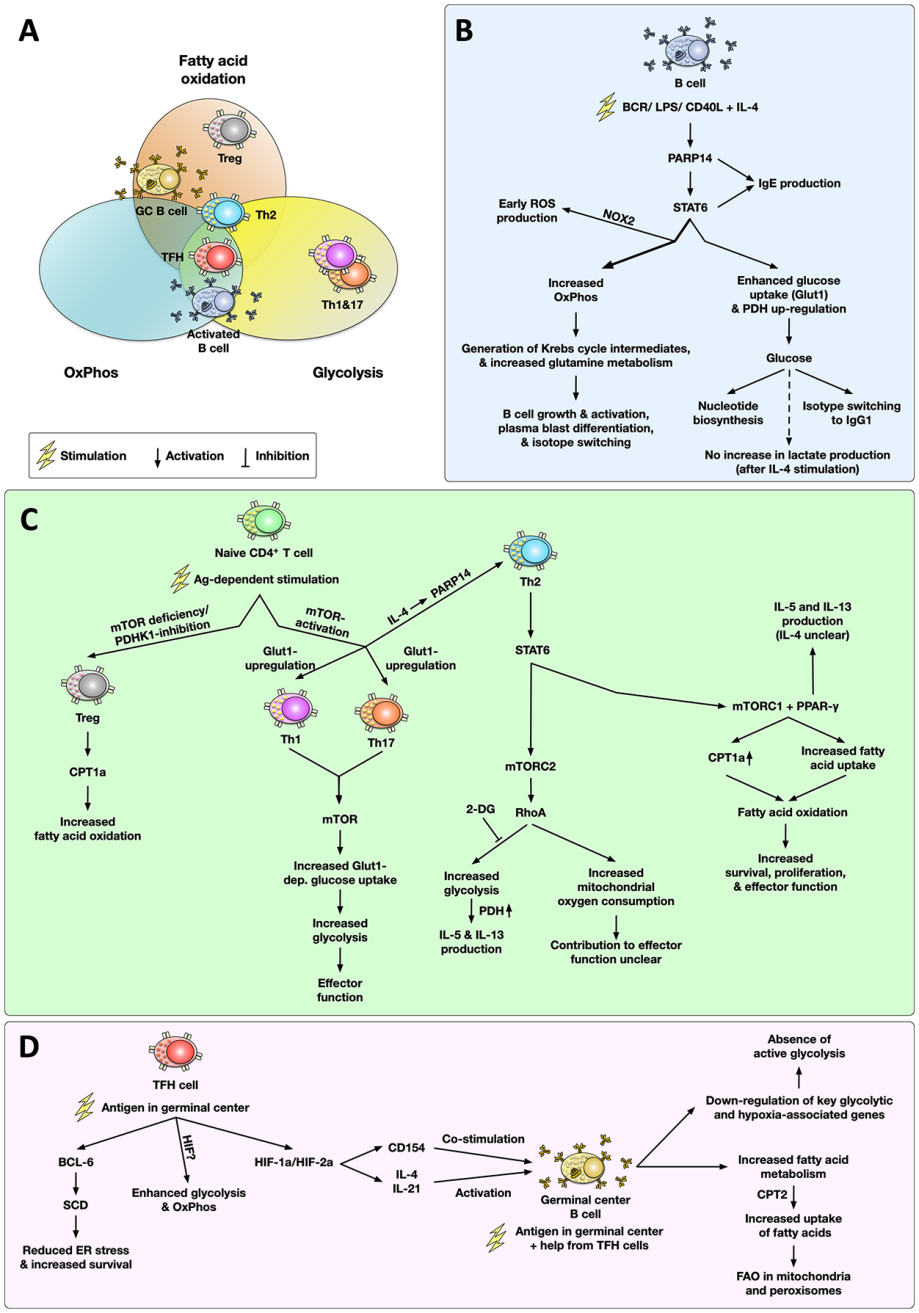
Metabolic phenotype and main signaling pathways associated with the activation of T and B cells in allergies. Cell types are grouped within the metabolic pathways (glycolysis, oxidative phosphorylation (OxPhos), fatty acid oxidation (FAO)) according to the published and discussed literature **(A)**. Upon activation, IL-4-stimulated B cells undergo complex metabolic changes, including a poly ADP-ribose polymerase 14 (PARP14)-dependent increase in glucose consumption driving both nucleotide synthesis and IgG1 production as well as high rates of OxPhos and glutamine metabolism, which promote B cell activation, plasmablast differentiation, and isotype switching **(B)**. In naïve CD4^+^ T cells antigen-dependent stimulation results in a mechanistic target of rapamycin (mTOR)-dependent T helper cell differentiation. While inhibition of mTOR results in differentiation of regulatory T cells (Treg) with a predominant carnitine palmitoyl transferase 1a (CPT1a)-dependent increase in FAO, mTOR activation is critically important for either Th1, Th2, Th17 differentiation. In both Th1 and Th17 cells, mTOR activation drives a glycolytic phenotype. In Th2 cells, mTOR complex 2 (mTORC2) promotes a RhoA-dependent increase in glycolysis (which was shown to contribute to IL-5 and IL-13 production) and an increase in mitochondrial oxygen consumption (whose contribution to Th2 effector function is currently unclear). Moreover, activation of mTORC1 and peroxisome proliferator-activated receptor gamma (PPAR-γ) in Th2 cells promotes fatty acid uptake and oxidation which fuels Th2 cell survival, proliferation, and effector function **(C)**. In germinal centers (GC), follicular T helper cells (TFH) HIF-1/2a-dependently promote the activation of germinal center B cells *via* CD154-dependent co-stimulation and the production of the switching cytokines IL-4 and IL-21 **(D)**. Here, TFH cells display both enhanced glycolysis and OxPhos while using the lipid metabolism enzyme stearoyl-CoA desaturase (SCD) to reduce ER stress and increase survival. In contrast to other cells types, antigen-activated GC B cells mainly rely on FAO for energy generation while suppressing glycolytic genes. For more detailed information see text. OxPhos, oxidative phosphorylation; FAO, fatty acid oxidation; BCR, B cell receptor; LPS, lipopolysaccharide; Glut1, glucose transporter 1; TCR, T cell receptor; 2-DG, 2-deoxy glucose; mTOR(C1/2), mechanistic target of rapamycin complex 1/2; PDH(K1), pyruvate dehydrogenase (kinase 1); LDH, lactate dehydrogenase; Treg, regulatory T cell; CPT1a/2, carnitine palmitoyl transferase 1a/2; PARP14, Poly ADP-ribose polymerase 14; PPAR-γ, peroxisome proliferator-activated receptor gamma; ROS, reactive oxygen species; STAT6, signal transducer and activator of transcription 6; NOX2, NADPH oxidase 2; BCL-6, B-cell lymphoma 6 protein; GC, germinal center; TFH cell, follicular T helper cell; SCD, stearoyl-CoA desaturase; HIF-1/2a, hypoxia inducible factor 1/2a.

Currently, the connection between T and B cell metabolism and either the development or maintenance of allergies is not completely understood. Therefore, this mini review will focus on the metabolism of IL-4-activated B cells and Th2 cells (the pathways and metabolic phenotypes associated with the activation of B and T cells discussed in this review are depicted in **Figure 2**).

### Immune Metabolic Changes Associated With IL-4-Activated B Cells

The available literature suggests that the metabolic changes associated with activated B cells are complex and strongly depend on the activating stimulus.

Upon either B cell receptor (BCR) crosslinking (28) or LPS-stimulation (29), activated B cells demonstrate enhanced glucose uptake, shown by e.g. increased glucose transporter 1 (Glut1) expression (**Figures 2A, B**). Furthermore, Caro-Maldano et al. showed stimulation of B cells with LPS leading to both increased ECAR-, and enhanced basal and maximal OCR-values (29).

In resting B cells, BCR-stimulation triggered NADPH oxidase 2 (NOX2)-dependent ROS-production in the first hour, which later switched to mitochondrial respiration (**Figure 2B**) (30), suggesting a connection between B cell oxygen consumption and ROS-production in pathogen clearance.

Haniuda et al. demonstrated that stimulation of B cells with IL-4 enhanced mitochondrial metabolism characterized by an accumulation of α-ketoglutarate and further promoted *Bcl6* gene expression, which affected B cell proliferation and differentiation (31). Moreover, in strongly proliferating B cells stimulated with CD40L and IL-4, Waters and colleagues observed the generation of Krebs cycle intermediates, an upregulated nucleotide biosynthesis fueled by glucose, and an increase in OxPhos, suggesting enhanced mitochondrial activity (**Figure 2B**) (32). In line with the observation that glycolysis mainly fueled nucleotide biosynthesis in IL-4-stimulated B cells, secretion of the glycolytic end-product lactate was shown to be enhanced in B cells stimulated with LPS, while being undetectable after IL-4 stimulation (33), which suggests that the respective stimulus strongly influences the metabolism of B cells and therefore the resulting accumulation of metabolites.

Mechanistically, essential roles of poly ADP-ribose polymerase (PARP)14 and signal transducer and activator of transcription 6 (STAT6) were demonstrated for IL-4 induced glycolysis in B cell cultures *in vitro* (33): PARP14 was shown to act as a transcriptional switch promoting both STAT6-binding to target genes and IL-4-dependent transcription (34). Furthermore, Cho et al. showed that IL-4 enhanced mitochondrial respiration only in wild type (WT, C57BL/6 for PARP14- and B6/129-intercrossed animals for STAT6-deficient mice) but not in either PARP14- or STAT6-deficient B cells (33). Additionally, they found IL-4 to increase expression levels of the Krebs cycle enzyme citrate synthase in IL-4-stimulated WT C57BL/6- but not in PARP14-deficient B cells (33). Furthermore, IL-4-induced PDH gene expression was reduced in PARP14-deficient B cells (33), suggesting PARP14 to be an essential factor in IL-4-dependent metabolic regulation of B cells (**Figure 2B**). PARP14 and STAT6 were also shown to be important for IgE production by IL-4-stimulated B cells, as both STAT6^−/−^ and PARP14^−/−^ mice showed decreased anti-ovalbumin IgE production from ovalbumin-sensitized mice compared to WT controls (C57BL/6 for PARP14- and B6/129-intercrossed for STAT6-deficient mice) (**Figure 2B**) (35).

Interestingly, B cell differentiation depended on mitochondrial metabolism, as inhibition of OxPhos resulted in decreased size and reduced MHCII- and CD86 expression *in vitro* (32). Moreover, the restriction of glutamine and OxPhos-inhibition also led to a lack of class switch recombination and plasma blast differentiation (**Figure 2B**) (32). In this experimental setting, glucose restriction only impaired isotype switching to IgG1 but had little to no effect on either B cell size, B cell activation (MHCII and CD86), or B cell differentiation (CD138 and frequency of B220^+^Fas^+^GL7^+^) (**Figure 2B**) (32). These results underline the importance of both glutamine metabolism and mitochondrial respiration for B cell development and function.

Germinal centers (GC) are specialized microenvironments within secondary lymphoid tissues in which antigen-activated B cells undergo clonal expansion, immunoglobulin class switching, and affinity maturation (36). Within GC, B cells that express high-affinity BCRs are positively selected by follicular T helper cells (TFH cells, see below).

Upon activation, GC B cells are among the fastest dividing cells in our body. Consequently, GC B cells are under a considerable proliferative stress when undergoing clonal expansion, somatic hypermutation, and antibody production. Zhu et al. recently showed the importance of the Akt pathway in regulating the metabolism of GC B cells. Here, the Akt isoforms Akt1 and Akt2 were shown to be essential for GC formation and maintenance, affinity maturation, and antibody production (36). These defects in GC B cells were due to reduced survival and proliferation as well as impaired mitochondrial and metabolic fitness (36). Here, restoration of proliferation and energy production *via* CD40-dependent T cell help was shown to rescue GC responses in AKT1/2-deficient animals (**Figure 2D**) (36).

The local micromilieu of GC was shown to by hypoxic (37). Consequently, GC B cells were thought to rely on oxygen-independent glycolysis for energy generation.

This assumption was recently challenged by Weisel and colleagues. Using untouched bead-based purification, Weisel investigated *ex vivo* bona fide GC B cells and appropriate *in vivo*–generated proliferating B cell controls to evaluate GC B cell metabolism (38). In their experiments, Weisel et al. could show that GC B cells displayed very low levels of extracellular acidification as well as little glycolytic reserve, indicating the absence of active glycolysis (38). Furthermore, GC B cells had higher levels of oxygen consumption than either resting naïve B cells or activated T cells, which could be inhibited by the carnitine palmitoyltransferase 1 (CPT1) inhibitor Etomoxir (38).

In line with these results, GC B cells were shown to oxidize endogenous and exogenous fatty acids while uptake of exogenous glucose was shown to be minimal (**Figure 2D**) (38). Uptake of fatty acids was paralleled by higher expression levels of the fatty acid transporter CD36, and carbon tracing experiments showed the GC B cells to predominantly generate acetyl-CoA from exogenous palmitate instead of glucose (38). Interestingly, fatty acid oxidation of GC B cells was shown to occur in both mitochondria and peroxisomes (**Figure 2D**) (38).

Notably, even upon inhibition of fatty acid oxidation, GC B cells did not switch to glycolytic metabolism, suggesting that GC B cells inherently repress glycolysis (38). In line with this, transcriptomic analyses showed a significant downregulation of several key glycolytic enzymes and hypoxia-associated genes (including HIF-1a) in GC B cells compared to activated non-GC B cells, suggesting that GC B cell gene expression does not reflect functional hypoxia (**Figure 2D**) (38).

Finally, genetic knock down of the mitochondrial fatty acid transporter CPT2 significantly reduced the numbers of GC B cells *in vivo*, showing the functional importance of FAO for GC B cells (38).

Taken together, these results show that highly proliferative GC B cells, in contrast to other B cell types (which employ both aerobic glycolysis and FAO), critically rely on FAO but not glucose metabolism for their function. Weisel and colleagues speculated that this prominent reliance on FAO of GC B cells might be an adaptation to their special niche: here, GC B cells acquire their energy in the form of fat derived from both the cell membranes and fat energy stores of cells dying within the germinal center (38).

In summary, activated B cells undergo complex metabolic changes, including increased glucose consumption and high rates of OxPhos, glutamine metabolism, nucleotide biosynthesis, and fatty acid oxidation. Interestingly, while GC B cells primarily relied on fatty acid oxidation for their energy generation, the observed increase in nucleotide biosynthesis in non-GC B cells was fueled by glucose (**Figure 2B**). Finally, B cells treated with Th2 cytokine IL-4 (and CD40L) display a reduced reliance on lactate production, but increased levels of mitochondrial respiration (**Figure 2A**), showing that B cells change their metabolic profile during allergic responses.

### Immune Metabolic Changes Associated With the Activation of Th2 and TFH Cells

The acute activation of T cells, their differentiation into CD8^+^ and CD4^+^ T effector cells, as well as the formation of memory T cells are all paralleled by distinct changes in cellular metabolism (**Figure 2A**).

Upon T cell receptor (TCR)-activation, the differentiation into effector CD4^+^ T helper cells (Th1, Th2, Th17, but not Tregs) is associated with an upregulation of Glut1 expression and a highly glycolytic phenotype *in vitro* (**Figure 2C**) (39). Furthermore, the mechanistic target of rapamycin (mTOR) was shown to be a major regulator of Th1-, Th2-, and Th17-differentiation, while mTOR-deficiency instead promoted the differentiation of Tregs both *in vitro* and *in vivo* (**Figure 2C**) (40). Moreover, a recent review by Pelgrom and Everts suggested the GTPase RhoA, a downstream target of mTOR complex 2 (mTORC2), promoted both higher glycolytic rates and mitochondrial oxygen consumption in Th2 cells compared to either Th1 or Th17 cells (**Figure 2C**) [reviewed in (10)]. Glycolysis was also shown to be important for Th2 cytokine production: Tibbitt et al. demonstrated that blocking glycolysis by administering 2-deoxyglucose (2-DG) in a house dust mite (HDM) mouse allergy model could reduce the expression of IL-13 and IL-5 (**Figure 2C**) (41). Moreover, Yagi and colleagues also found that inhibition of PDH could suppress the generation of IL-5- and IL-13-producing Th2 cells (**Figure 2C**) (42). Taken together, these studies indicate that glucose metabolism is critically involved in both the differentiation of Th2 cells and their effector function.

Besides glucose metabolism, the usage of fatty acids was also found to be different in Th2 cells from asthmatic individuals: research from Seumois and colleagues found higher expression of carnitine palmitoyltransferase I isoform a (CPT1a), a mitochondrial enzyme responsible for long-chain fatty acid oxidation, in asthmatic patients compared to healthy controls (43). Interestingly, CPT1a could further increase both the effector function and survival rate of Th2 cells (**Figure 2C**) (43). Mechanistically, Angela et al. demonstrated that mTORC1 and peroxisome proliferator-activated receptor gamma (PPAR-γ, a transcription factor) play essential roles in the lipid metabolism of Th2 cells (44), regulating fatty acid uptake by Th2 cells, and influencing the proliferation of Th2 cells (**Figure 2C**) (45). Also, compared to naïve T cells, memory Th2 cells took up more extracellular fatty acids after antigenic stimulation (45). Therefore, FAO may also contribute to the effector function of Th2 cells. Here, more work is needed to study the contribution of Th2 cell fatty acid metabolism in allergic inflammation.

IL-4 is an important Th2 cell effector cytokine in both mice and humans, and IL-4-stimulated Th2 cells were shown to activate STAT6 (**Figure 2C**) (46, 47). In accordance with the results for PARP14-dependent STAT6-induced gene transcription in IL-4-stimulated B cells (see above) (34), PARP14 was shown to regulate Th2 differentiation *in vitro* (48): In an OVA-based allergic airway disease model, PARP14-knock out mice showed reduced lung pathology, reduced production of Th2 cytokines, and lower IgE levels in serum compared to WT mice (48).

PPAR-γ is not only involved in fatty acid uptake by Th2 cells, but is also suggested to be induced in Th2 cells by IL-4R ligation and subsequent STAT6 activation (9), as has been reported for macrophages (49, 50) and dendritic cells (49). However, the effect of PPAR-γ on Th2 cell-related IL-4 production is controversial and seems to be assay-dependent (9): previous studies showed a striking reduction in the frequencies of IL-4 expressing cells in PPAR-γ-deficient mice sensitized with HDM extract (51), while others reported IL-4 producing cells to remain unchanged in PPAR-γ-deficient mice (52). By contrast, Park and colleagues showed that PPAR-γ-deficient T cells secreted higher levels of IL-4 upon anti-CD3/CD28 stimulation (53). Here, the impact of PPAR-γ on IL-5- and IL-13-production was more clear, as Th2 cells from PPAR-γ^-/-^ mice failed to produce either IL-5- or IL-13 (**Figure 2C**) (51, 52).

TFH cells are specialized CD4^+^ T helper cells that regulate the formation of germinal centers and the production of class-switched high-affinity antibodies (54). TFH cells interact with GC B cells *via* the expression of co-stimulatory signals (CD154 on TFH cells interacting with CD40 on GC B cells) and cytokines such as IL-4 and IL-21 (55).

Using different metabolic inhibitors in an *in vivo* model of influenza infection, Dong et al. described both HIF-1a-dependent glycolysis and OxPhos to be important for TFH cell differentiation and GC responses (56). In line with this, Cho et al. described both HIF-1a and HIF-2a in CD4^+^ T cells to regulate (I) the expression of CD154 on TFH cells which is critical for CD40-mediated co-stimulation of GC B cells, (II) the production of cytokines necessary to induce isotype switching in activated B cells, and (III) metabolic reprogramming of activated T cells towards glycolysis (**Figure 2D**) (57). Accordingly, HIF-1a depletion in CD4^+^ T cells reduced the frequencies of antigen-specific GC B cells, TFH cells, and overall levels of antigen-specific antibodies after immunization with sheep red blood cells (57). Cho et al. proposed a model in which HIF-expression shifts the balance between follicular regulatory and helper T cells while also regulating their metabolic state, numbers of follicular helper cells, as well as the molecules they express to promote antibody production (57).

Moreover, Son et al. recently described a non-redundant role of lipid metabolism in regulating TFH maintenance and GC B cell responses. They found both human and mouse TFH cells to B-cell lymphoma 6 protein (BCL-6)-dependently express higher levels of stearoyl-CoA desaturase (SCD). Inhibition of SCD reduced both TFH and GC responses *in vivo* following immunization with the X31 influenza strain (58). Here, inhibition of SCD promoted the expression of ER stress genes and enhanced TFH cell apoptosis *in vitro* and *in vivo* (**Figure 2D**) (58). In contrast, SCD inhibition had no effects on either the total number of either CD4^+^ T cells or B cells or the production of IFN-γ, suggesting that SCD inhibition selectively impaired TFH but not Th1 cell formation (58).

Taken together, metabolically, Th2 cells seem to mainly depend on glycolysis and FAO (**Figure 2A**). Here, the discussed mTOR-, PARP14-, and PPAR-γ-pathways are reported to both regulate Th2 cell metabolism and shape their effector functions (**Figure 2C**). On the other hand, TFH cells rely on HIF-1a-dependent glycolysis and OxPhos with lipid metabolism being involved in the promoting of TFH cell survival.

## Conclusions

Our review discusses evidence that, depending on the stimulus, the metabolic changes observed in B cells are complex and include increases in glucose consumption, OxPhos, glutamine metabolism, and nucleotide biosynthesis. On the other hand, Th2-biased effector T cells rely more on glycolysis and fatty acid metabolism, which are also closely linked to their effector functions.

Unraveling the metabolic changes in allergic contexts and their contribution to allergic inflammation will allow us to better understand both allergic pathology and develop new treatment options in the future.

## Author Contributions

All authors listed have made a substantial, direct, and intellectual contribution to the work and approved it for publication.

## Funding

This work was in part funded by the budget of the Paul-Ehrlich-Institut, Langen, Germany. AG was funded by the German Research Foundation (DFG SCHU2951/4). Y-JL was funded by the German Research Foundation (DFG SCHE637/4).

## Conflict of Interest

The authors declare that the research was conducted in the absence of any commercial or financial relationships that could be construed as a potential conflict of interest.

## Publisher’s Note

All claims expressed in this article are solely those of the authors and do not necessarily represent those of their affiliated organizations, or those of the publisher, the editors and the reviewers. Any product that may be evaluated in this article, or claim that may be made by its manufacturer, is not guaranteed or endorsed by the publisher.

## Abbreviations

2-DG, 2-deoxyglucose; APC, antigen presenting cells; BCL-6, B-cell lymphoma 6 protein; BCR, B cell receptor; CPT1(a), carnitine palmitoyl transferase; ECAR, extracellular acidification rate; FAO, fatty acid oxidation; GC, germinal centers; GDH, glutamate dehydrogenase; Glut1, glucose transporter 1; HDM, house dust mite; HIF-1a, hypoxia inducible factor 1a; IDH, isocitrate dehydrogenase; LPS, lipopolysaccharide; mTOR, mechanistic target of rapamycin; mTORC(1/2), mTOR complex 1/2; NO, nitric oxide; NOX2, NADPH oxidase 2; OCR, oxygen consumption rate; OxPhos, oxidative phosphorylation; PARP14, Poly ADP-ribose polymerase 14; PDH, pyruvate dehydrogenase; PDHK(1), pyruvate dehydrogenase kinase (1); PPAR-γ, peroxisome proliferator-activated receptor gamma; ROS, reactive oxygen species; SCD, stearoyl-CoA desaturase; STAT6, signal transducer and activator of transcription 6; TCR, T cell receptor; TFH cell, follicular T helper cell; Treg, regulatory T cells; WT, wild type.

## References

[1] Pelaia G Vatrella A Busceti MT Gallelli L Calabrese C Terracciano R. Cellular Mechanisms Underlying Eosinophilic and Neutrophilic Airway Inflammation in Asthma. Mediators Inflamm (2015) 2015:879783–8. doi: 10.1155/2015/879783 PMC4386709 25878402

[2] Flöistrup H Swartz J Bergström A Alm JS Scheynius A van Hage M. Allergic Disease and Sensitization in Steiner School Children. J Allergy Clin Immunol (2006) 117:59–66. doi: 10.1016/j.jaci.2005.09.039 16387585

[3] Larché M Akdis CA Valenta R. Immunological Mechanisms of Allergen-Specific Immunotherapy. Nat Rev Immunol (2006) 6:761–71. doi: 10.1038/nri1934 16998509

[4] Jung J Zeng H Horng T. Metabolism as a Guiding Force for Immunity. Nat Cell Biol (2019) 21:85–93. doi: 10.1038/s41556-018-0217-x 30602764

[5] Gaber T Strehl C Buttgereit F. Metabolic Regulation of Inflammation. Nat Rev Rheumatol (2017) 13:267–79. doi: 10.1038/nrrheum.2017.37 28331208

[6] O’Neill LAJ Kishton RJ Rathmell J. a Guide to Immunometabolism for Immunologists. Nat Rev Immunol (2016) 16:553–65. doi: 10.1038/nri.2016.70 PMC5001910 27396447

[7] Goretzki A Lin Y-J Schülke S. Immune Metabolism in Allergies, Does It Matter?-A Review of Immune Metabolic Basics and Adaptations Associated With the Activation of Innate Immune Cells in Allergy. Allergy (2021) 76(11):3314–3331. doi: 10.1111/all.14843 33811351

[8] Obeso D Mera-Berriatua L Rodríguez-Coira J Rosace D Fernández P Martín-Antoniano IA. Multi-Omics Analysis Points to Altered Platelet Functions in Severe Food-Associated Respiratory Allergy. Allergy (2018) 73:2137–49. doi: 10.1111/all.13563 30028518

[9] Stark JM Tibbitt CA Coquet JM. The Metabolic Requirements of Th2 Cell Differentiation. Front Immunol (2019) 10:2318. doi: 10.3389/fimmu.2019.02318 31611881 PMC6776632

[10] Pelgrom LR Everts B. Metabolic Control of Type 2 Immunity. Eur J Immunol (2017) 47:1266–75. doi: 10.1002/eji.201646728 28661041

[11] Boothby M Rickert RC. Metabolic Regulation of the Immune Humoral Response. Immunity (2017) 46:743–55. doi: 10.1016/j.immuni.2017.04.009 PMC5640164 28514675

[12] Michaeloudes C Bhavsar PK Mumby S Xu B Hui CKM Chung KF. Role of Metabolic Reprogramming in Pulmonary Innate Immunity and Its Impact on Lung Diseases. J Innate Immun (2020) 12:31–46. doi: 10.1159/000504344 31786568 PMC6959099

[13] van der Bliek AM Sedensky MM Morgan PG. Cell Biology of the Mitochondrion. Genetics (2017) 207:843–71. doi: 10.1534/genetics.117.300262 PMC5676242 29097398

[14] Kim J Tchernyshyov I Semenza GL Dang CV. HIF-1-Mediated Expression of Pyruvate Dehydrogenase Kinase: A Metabolic Switch Required for Cellular Adaptation to Hypoxia. Cell Metab (2006) 3:177–85. doi: 10.1016/j.cmet.2006.02.002 16517405

[15] Gatenby RA Gillies RJ. Why do Cancers Have High Aerobic Glycolysis? Nat Rev Cancer (2004) 4:891–9. doi: 10.1038/nrc1478 15516961

[16] Jones N Vincent EE Felix LC Cronin JG Scott LM Hole PS. Interleukin-5 Drives Glycolysis and Reactive Oxygen Species-Dependent Citric Acid Cycling by Eosinophils. Allergy (2020) 75:1361–70. doi: 10.1111/all.14158 31856334

[17] Jones W Bianchi K. Aerobic Glycolysis: Beyond Proliferation. Front Immunol (2015) 6:227. doi: 10.3389/fimmu.2015.00227 26029212 PMC4432802

[18] Warburg O. On the Origin of Cancer Cells. Science (1956) 123:309–14. doi: 10.1126/science.123.3191.309 13298683

[19] Ros S Schulze A. Balancing Glycolytic Flux: The Role of 6-Phosphofructo-2-Kinase/Fructose 2,6-Bisphosphatases in Cancer Metabolism. Cancer Metab (2013) 1:8. doi: 10.1186/2049-3002-1-8 24280138 PMC4178209

[20] Lunt SY Vander Heiden MG. Aerobic Glycolysis: Meeting the Metabolic Requirements of Cell Proliferation. Annu Rev Cell Dev Biol (2011) 27:441–64. doi: 10.1146/annurev-cellbio-092910-154237 21985671

[21] Kelly B O’Neill LAJ. Metabolic Reprogramming in Macrophages and Dendritic Cells in Innate Immunity. Cell Res (2015) 25:771–84. doi: 10.1038/cr.2015.68 PMC4493277 26045163

[22] Loftus RM Finlay DK. Immunometabolism: Cellular Metabolism Turns Immune Regulator. J Biol Chem (2016) 291:1–10. doi: 10.1074/jbc.R115.693903 26534957 PMC4697146

[23] Caslin HL Taruselli MT Haque T Pondicherry N Baldwin EA Barnstein BO. Inhibiting Glycolysis and ATP Production Attenuates IL-33-Mediated Mast Cell Function and Peritonitis. Front Immunol (2018) 9:3026. doi: 10.3389/fimmu.2018.03026 30619366 PMC6305324

[24] Ryan DG O’Neill LAJ. Krebs Cycle Rewired for Macrophage and Dendritic Cell Effector Functions. FEBS Lett (2017) 591:2992–3006. doi: 10.1002/1873-3468.12744 28685841

[25] Ryan DG O’Neill LAJ. Krebs Cycle Reborn in Macrophage Immunometabolism. Annu Rev Immunol (2020) 38:289–313. doi: 10.1146/annurev-immunol-081619-104850 31986069

[26] Jha AK Huang SC-C Sergushichev A Lampropoulou V Ivanova Y Loginicheva E. Network Integration of Parallel Metabolic and Transcriptional Data Reveals Metabolic Modules That Regulate Macrophage Polarization. Immunity (2015) 42:419–30. doi: 10.1016/j.immuni.2015.02.005 25786174

[27] Arts RJW Novakovic B Ter Horst R Carvalho A Bekkering S Lachmandas E. Glutaminolysis and Fumarate Accumulation Integrate Immunometabolic and Epigenetic Programs in Trained Immunity. Cell Metab (2016) 24:807–19. doi: 10.1016/j.cmet.2016.10.008 PMC5742541 27866838

[28] Doughty CA Bleiman BF Wagner DJ Dufort FJ Mataraza JM Roberts MF. Antigen Receptor-Mediated Changes in Glucose Metabolism in B Lymphocytes: Role of Phosphatidylinositol 3-Kinase Signaling in the Glycolytic Control of Growth. Blood (2006) 107:4458–65. doi: 10.1182/blood-2005-12-4788 PMC1895797 16449529

[29] Caro-Maldonado A Wang R Nichols AG Kuraoka M Milasta S Sun LD. Metabolic Reprogramming Is Required for Antibody Production That is Suppressed in Anergic But Exaggerated in Chronically BAFF-Exposed B Cells. J Immunol (2014) 192:3626–36. doi: 10.4049/jimmunol.1302062 PMC3984038 24616478

[30] Wheeler ML Defranco AL. Prolonged Production of Reactive Oxygen Species in Response to B Cell Receptor Stimulation Promotes B Cell Activation and Proliferation. J Immunol (2012) 189:4405–16. doi: 10.4049/jimmunol.1201433 PMC3515638 23024271

[31] Haniuda K Fukao S Kitamura D. Metabolic Reprogramming Induces Germinal Center B Cell Differentiation Through Bcl6 Locus Remodeling. Cell Rep (2020) 33:108333. doi: 10.1016/j.celrep.2020.108333 33147467

[32] Waters LR Ahsan FM Wolf DM Shirihai O Teitell MA. Initial B Cell Activation Induces Metabolic Reprogramming and Mitochondrial Remodeling. iScience (2018) 5:99–109. doi: 10.1016/j.isci.2018.07.005 30240649 PMC6123864

[33] Cho SH Ahn AK Bhargava P Lee C-H Eischen CM McGuinness O. Glycolytic Rate and Lymphomagenesis Depend on PARP14, an ADP Ribosyltransferase of the B Aggressive Lymphoma (BAL) Family. Proc Natl Acad Sci USA (2011) 108:15972–7. doi: 10.1073/pnas.1017082108 PMC3179111 21911376

[34] Mehrotra P Riley JP Patel R Li F Voss L Goenka S. PARP-14 Functions as a Transcriptional Switch for Stat6-Dependent Gene Activation. J Biol Chem (2011) 286:1767–76. doi: 10.1074/jbc.M110.157768 PMC3023471 21081493

[35] Cho SH Raybuck A Wei M Erickson J Nam KT Cox RG. Boothby M. B Cell-Intrinsic and -Extrinsic Regulation of Antibody Responses by PARP14, an Intracellular (ADP-Ribosyl)Transferase. J Immunol (2013) 191:3169–78. doi: 10.4049/jimmunol.1301106 PMC3770464 23956424

[36] Zhu Z Shukla A Ramezani-Rad P Apgar JR Rickert RC. The AKT Isoforms 1 and 2 Drive B Cell Fate Decisions During the Germinal Center Response. Life Sci Alliance (2019) 2:e201900506. doi: 10.26508/lsa.201900506 31767615 PMC6878223

[37] Cho SH Raybuck AL Stengel K Wei M Beck TC Volanakis E. Germinal Centre Hypoxia and Regulation of Antibody Qualities by a Hypoxia Response System. Nature (2016) 537:234–8. doi: 10.1038/nature19334 PMC5161594 27501247

[38] Weisel FJ Mullett SJ Elsner RA Menk AV Trivedi N Luo W. Germinal Center B Cells Selectively Oxidize Fatty Acids for Energy While Conducting Minimal Glycolysis. Nat Immunol (2020) 21:331–42. doi: 10.1038/s41590-020-0598-4 PMC7112716 32066950

[39] Michalek RD Gerriets VA Jacobs SR Macintyre AN MacIver NJ Mason EF. Cutting Edge: Distinct Glycolytic and Lipid Oxidative Metabolic Programs are Essential for Effector and Regulatory CD4+ T Cell Subsets. J Immunol (2011) 186:3299–303. doi: 10.4049/jimmunol.1003613 PMC3198034 21317389

[40] Delgoffe GM Kole TP Zheng Y Zarek PE Matthews KL Xiao B. The Mtor Kinase Differentially Regulates Effector and Regulatory T Cell Lineage Commitment. Immunity (2009) 30:832–44. doi: 10.1016/j.immuni.2009.04.014 PMC2768135 19538929

[41] Tibbitt CA Stark JM Martens L Ma J Mold JE Deswarte K. Single-Cell RNA Sequencing of the T Helper Cell Response to House Dust Mites Defines a Distinct Gene Expression Signature in Airway Th2 Cells. Immunity (2019) 51:169–84.e5. doi: 10.1016/j.immuni.2019.05.014 31231035

[42] Yagi Y Kuwahara M Suzuki J Imai Y Yamashita M. Glycolysis and Subsequent Mevalonate Biosynthesis Play an Important Role in Th2 Cell Differentiation. Biochem Biophys Res Commun (2020) 530:355–61. doi: 10.1016/j.bbrc.2020.08.009 32800342

[43] Seumois G Zapardiel-Gonzalo J White B Singh D Schulten V Dillon M. Transcriptional Profiling of Th2 Cells Identifies Pathogenic Features Associated With Asthma. J Immunol (2016) 197:655–64. doi: 10.4049/jimmunol.1600397 PMC4936908 27271570

[44] Hernandez-Quiles M Broekema MF Kalkhoven E. Ppargamma in Metabolism, Immunity, and Cancer: Unified and Diverse Mechanisms of Action. Front Endocrinol (Lausanne) (2021) 12:624112. doi: 10.3389/fendo.2021.624112 33716977 PMC7953066

[45] Angela M Endo Y Asou HK Yamamoto T Tumes DJ Tokuyama H. Fatty Acid Metabolic Reprogramming *via* Mtor-Mediated Inductions of Pparγ Directs Early Activation of T Cells. Nat Commun (2016) 7:13683. doi: 10.1038/ncomms13683 27901044 PMC5141517

[46] Kaplan MH Schindler U Smiley ST Grusby MJ. Stat6 Is Required for Mediating Responses to IL-4 and for Development of Th2 Cells. Immunity (1996) 4:313–9. doi: 10.1016/s1074-7613(00)80439-2 8624821

[47] Elo LL Järvenpää H Tuomela S Raghav S Ahlfors H Laurila K. Genome-Wide Profiling of Interleukin-4 and STAT6 Transcription Factor Regulation of Human Th2 Cell Programming. Immunity (2010) 32:852–62. doi: 10.1016/j.immuni.2010.06.011 20620947

[48] Mehrotra P Hollenbeck A Riley JP Li F Patel RJ Akhtar N. Poly (ADP-Ribose) Polymerase 14 and Its Enzyme Activity Regulates T(H)2 Differentiation and Allergic Airway Disease. J Allergy Clin Immunol (2013) 131:521–31. doi: 10.1016/j.jaci.2012.06.015 PMC3502685 22841009

[49] Szanto A Balint BL Nagy ZS Barta E Dezso B Pap A. STAT6 Transcription Factor is a Facilitator of the Nuclear Receptor Pparγ-Regulated Gene Expression in Macrophages and Dendritic Cells. Immunity (2010) 33:699–712. doi: 10.1016/j.immuni.2010.11.009 21093321 PMC3052437

[50] Daniel B Nagy G Czimmerer Z Horvath A Hammers DW Cuaranta-Monroy I. The Nuclear Receptor Pparγ Controls Progressive Macrophage Polarization as a Ligand-Insensitive Epigenomic Ratchet of Transcriptional Memory. Immunity (2018) 49:615–26.e6. doi: 10.1016/j.immuni.2018.09.005 30332629 PMC6197058

[51] Nobs SP Natali S Pohlmeier L Okreglicka K Schneider C Kurrer M. Pparγ in Dendritic Cells and T Cells Drives Pathogenic Type-2 Effector Responses in Lung Inflammation. J Exp Med (2017) 214:3015–35. doi: 10.1084/jem.20162069 PMC5626395 28798029

[52] Chen T Tibbitt CA Feng X Stark JM Rohrbeck L Rausch L. Ppar-γ Promotes Type 2 Immune Responses in Allergy and Nematode Infection. Sci Immunol (2017) 2:eaal5196. doi: 10.1126/sciimmunol.aal5196 28783701

[53] Park H-J Kim D-H Choi J-Y Kim W-J Kim JY Senejani AG. Pparγ Negatively Regulates T Cell Activation to Prevent Follicular Helper T Cells and Germinal Center Formation. PLoS One (2014) 9:e99127. doi: 10.1371/journal.pone.0099127 24921943 PMC4055678

[54] Crotty S. T Follicular Helper Cell Differentiation, Function, and Roles in Disease. Immunity (2014) 41:529–42. doi: 10.1016/j.immuni.2014.10.004 PMC4223692 25367570

[55] Choi S-C Morel L. Immune Metabolism Regulation of the Germinal Center Response. Exp Mol Med (2020) 52:348–55. doi: 10.1038/s12276-020-0392-2 PMC7156389 32132626

[56] Dong L He Y Zhou S Cao Y Li Y Bi Y. Hif1α-Dependent Metabolic Signals Control the Differentiation of Follicular Helper T Cells. Cells (2019) 8:E1450. doi: 10.3390/cells8111450 31744227 PMC6912655

[57] Cho SH Raybuck AL Blagih J Kemboi E Haase VH Jones RG. Hypoxia-Inducible Factors in CD4+ T Cells Promote Metabolism, Switch Cytokine Secretion, and T Cell Help in Humoral Immunity. Proc Natl Acad Sci USA (2019) 116:8975–84. doi: 10.1073/pnas.1811702116 PMC6500120 30988188

[58] Son YM Cheon IS Goplen NP Dent AL Sun J. Inhibition of Stearoyl-Coa Desaturases Suppresses Follicular Help T- and Germinal Center B- Cell Responses. Eur J Immunol (2020) 50:1067–77. doi: 10.1002/eji.201948257 PMC8496969 32133634

